# Ambient geochemical baselines for trace elements in Chernozems—approximation of geochemical soil transformation in an agricultural area

**DOI:** 10.1007/s10661-018-7133-1

**Published:** 2018-12-15

**Authors:** Labaz Beata, Kabala Cezary, Waroszewski Jaroslaw

**Affiliations:** Wrocław University of Environmental and Life Sciences, Institute of Soil Science and Environmental Protection, Grunwaldzka 53, 50-357 Wrocław, Poland

**Keywords:** Chernozems, Loess, Trace elements, Ambient background, Geochemical baseline, Risk assessment

## Abstract

The legal regulatory/action levels of trace elements in soils are established at high concentrations, at which the crucial functions of soil are at risk or are eliminated. However, concentrations below these action levels, but above presumed natural levels, may also limit particular ecosystem services, including organic food production. Thus, defining the (ambient) background concentrations is an essential part of environmental or health risk assessment, e.g., on Chernozems, which are considered to be the most productive soils and ones that should be protected against all forms of contamination. Based on 28 profiles of chernozemic soils developed from loess in an agricultural region of SW Poland presumed to be free of industrial contamination, ambient geochemical baselines have been derived for Fe and six trace metals for four standardized soil layers, including the topsoil (plow layer) and parent material layers. The median values for the plow layer (1.89% for Fe, and 537, 49, 17, 14, and 26 mg kg^−1^ for Mn, Zn, Pb, Cu, and Ni, respectively) are lower than the values reported for other Chernozems in SE Poland/Europe/the world, and thus may serve as a general geochemical baseline for chernozemic soils developed from loess. The concentration of Cd, although lower than in other Chernozems around the world, is higher than in Ukrainian Chernozems and thus may serve as a local (or Central European) baseline only. The median concentrations of Fe, Cu, Mn, and Zn are very close to their concentrations in the Chernozem buried under the Neolithic kurgan. However, Pb and Cd concentrations are two times higher than in the buried soil, indicating the scale of general contamination of the topsoil horizons of arable soils. Concentrations of the elements under study, excluding Fe, in both the buried and surface soils are significantly higher in the topsoil layer compared to parent material (loess), and this justifies the separate baseline values for topsoil horizons, instead of background values derived universally for parent rock types. This is essential, in particular in soils texturally differentiated within profiles, where the subsoil material has a different origin and cannot be considered the parent material for topsoil horizons. Underlying or locally outcropped bedrock (e.g., serpentinite rocks) may naturally enhance the total concentration of trace elements in the entire soil profile by the addition of metal-rich regolith particles during the formation of surface covers, e.g., by eolian processes under periglacial conditions (Late Pleistocene). Such soils are naturally enriched with metals (with nickel in the case of serpentinite bedrock), cannot be considered contaminated, and thus require a separate legal treatment, including separate (or individually suited) background baselines for health risk assessments.

## Introduction

The content of trace elements in soil arouses great interest among farmers, ecologists, and biologists because soil, as an important medium for the circulation of elements in the environment, has a significant impact on the chemical composition of plants and therefore on the quality of food (Adriano 2001; Kabata-Pendias 2004; Karak et al. 2017). Many elements (e.g., Co, Cr, Cu, Fe, Mn, Ni, and Zn) are essential for plants as micronutrients or components of enzymes important in metabolic processes, nitrogen assimilation, transport through biological membranes, or the regulation of osmotic pressure in cells (Bruins et al. 2000; Hansda and Kumar 2017). For other elements (e.g., Cd, Hg, Pb, Au, Ag), no beneficial biological functions are recognized, or it has been found, on the contrary, that even small amounts of these disrupt biochemical processes and therefore are potentially toxic for living organisms (Pan et al. 2010; Seregin and Ivanov 2001; Kajka and Rutkowska 2018). Toxic impacts can also stem from these elements (Vodyanitskii 2013).

The amount of trace elements in soils is the result of their content derived from natural sources as well as from human activities (Alloway 2013). Among the natural factors, the key factor is the parent rock and those conditions which affect the intensity of rock weathering (Bonifacio et al. 2010; Orzechowski and Smólczynski 2010; Kierczak et al. 2016). In turn, anthropogenic influences can be direct, as in the intentional supply of elements along with mineral or organic fertilizers (Borui et al. 2017; Kabala et al. 2011; Schaecke et al. 2002), or indirect, as in fallout from metal-bearing airborne dust from industrial emissions (Ghayoraneh and Qishlaqi 2017; Komorowski and Szulc 2017; Kowalska et al. 2016). In heavily urbanized or industrial areas, the quantities of trace elements originating from anthropogenic sources can exceed the quantities originating from natural sources (Cannon and Horton 2009; Cheng et al. 2015; Wcisło et al. 2002; Tyszka et al. 2016).

The regulatory levels of trace elements (and other xenobiotics) have been approximated in most countries (Dung et al. 2013; Maliszewska-Kordybach and Smreczak 2003; Kabala et al. 2013; Mehr et al. 2017; Reimann and Garrett 2005). Metal levels have been differently defined and termed (e.g., action levels or maximum admissible concentration values), but, in general, the confirmation of metal content increases above those levels always results in the commencement of administrative procedures aimed at reducing the environmental or health risks (Karczewska and Kabala 2017; US EPA Interstate Technology and Regulatory Council 2008). Regulatory/action levels are established at a high metal concentration, one at which the crucial functions of soil are at risk or are eliminated, including food production, water filtration, and contaminant buffering (Ghayoraneh and Qishlaqi 2017; Medynska-Juraszek and Kabala 2012). A metal concentration in soil at the action level indicates an extreme state; however, various levels of soil contamination may occur that do not totally eliminate food production, but may limit particular soil/ecosystem services to various extents. In particular, “high quality food production” may require the best soil quality, including a “lack of soil contamination” with trace metals (Reganold and Wachter 2016), and this prompts the question as to the “natural,” “normal,” or “background” concentrations of metals in soils.

Originally, the term “geochemical background” was developed for exploration geochemistry and it was defined as a “normal” abundance of an element in rocks and barren earth materials. Any “anomalies,” i.e., a metal concentration that exceeds its “normal” concentration, have significant importance in the selection of sites for further exploration and potential metal ore exploitation (Hawkes and Webb 1963). The “threshold value” was, therefore, defined as the upper limit of normal background fluctuation, above which an anomalous concentration may be distinguished (Reimann et al. 2005). The geochemical background or the threshold values have importance in environmental sciences, including the soil sciences, in particular in relation to food production and health risk assessment, as they may provide reference values (1) to document the various levels of human-induced transformation of soil quality in relation to parent rock variability, organic matter content, land use, etc.; (2) to assess the relative impact of local contamination sources; (3) to interpret the results of large-scale or long-term monitoring series; and finally (4) to indicate those areas which are free of contamination and thus preferable for high-quality food production (Reimann et al. 2012; Salminen et al. 2005). In many studies, the element concentration in the parent rock horizon (C) of a soil profile has been considered the background for an entire soil profile. Based on this assumption, numerous enrichment or geoaccumulation indexes/factors have been derived (Mazurek et al. 2017; Woszczyk et al. 2018). However, natural pedogenic processes may significantly differentiate the vertical distribution of elements throughout the soil profile and may also enhance the content of elements in the topsoil horizons compared to the C layer (Gall et al. 2015; Sterckeman et al. 2006). Moreover, many soils have a lithologic discontinuity at various depths; thus, the bottom part of a soil profile may not represent the parent material for the topsoil (Waroszewski et al. 2015). Therefore, many researchers have argued that the element content in the parent material (C layer) has an indicative function only, whereas the “pedo-geochemical background” for topsoil layers should be separately derived (Baize and Sterckeman 2001; Zhao et al. 2007).

The ability to evaluate the “natural” background for soils is disputed, as soils have been impacted upon by human activity elsewhere since at least the industrial revolution. The term “ambient background” is sometimes suggested to describe the local modified “background” in areas impacted upon by human activity, where the metal concentrations in soils and sediments are slightly elevated, but do not generate any identifiable health or environmental risks (Reimann and Garrett 2005; Mikkonen et al. 2017, 2018). Finally, the term “geochemical baseline” has been suggested to approximate the present ambient background values (Wang et al. 2011). To allow comparisons, the baseline has to be given as a single number (“line”) instead of a range of values. Various “baselines” are commonly reported: (1) more restrictive statistical measures such as mean or median metal concentration, and (2) relative (expected) maximum levels, such as mean + 2 standard deviations or median + 2 median absolute deviations (Gałuszka 2007; Matschullat et al. 2000; Mikkonen et al. 2017).

Several methods are applied to estimate the background values for trace metals in soils and these are sometimes are designated “geochemical” or “statistical” (Matschullat et al. 2000). A “geochemical” attempt refers to pre-industrial archives (such as limnic or marine sediments, overbank and river sediments, cave sediments, or buried soils) that are correlated with a given target surface soil and normalized using respective soil properties, such as pH, texture, or organic matter (Wang et al. 2011). The “statistical” approaches approximate the ambient geochemical baseline based on soil samples collected in each area, where the baseline is required, but where the natural factors may be distinguished from the anthropogenic ones, using, e.g., regression analysis, fractal methods, probability plots, or outlier elimination (Dung et al. 2013; Filzmoser et al. 2005; Matschullat et al. 2000; Reimann et al. 2014; Zhou and Xia 2010). Among the latter methods, the iterative 2σ technique, more radical but less robust compared to the other tests, has become relatively popular (Gałuszka 2007; Mikkonen et al. 2017). The technique aims at defining the background by approaching a normal range, where the values beyond the mean ± 2σ interval are subsequently omitted (Matschullat et al. 2000). Another possible measure of the pollution degree or the natural concentration of trace elements in the topsoil layer is a determination of so-called enrichment factors, which are commonly applied due to their simple calculation (Kowalska et al. 2016, 2018; Mazurek et al. 2017). However, the enrichment factors are strongly criticized because they do not refer to the local or regional background for topsoil layers, and are unsuitable for soils with any lithological discontinuity present in the profile (Reimann and Caritat 2000; Sucharovà et al. 2012).

Chernozems are among the most productive soils in the world due to their thick humus horizon, the fact that they are structural and biologically active and enriched with organic matter and nutrients, as well as their silt-loamy texture, which is beneficial for water retention and supply (Novák et al. 2014; Šimansky and Jonczak 2016). Chernozems are recognized in many countries as high-priority soil resources, crucial for national food safety security policy (Chendev et al. 2017; Vysloužilová et al. 2016). Therefore, there is a common social expectation to protect Chernozems against degradation, in particular to safeguard these soils against contamination with trace metals (Kolesnikov et al. 1999; Minkina et al. 2008). The general statement that the concentration of metals in Chernozems is below the action levels, which is known from national soil monitoring programs, is unsatisfactory, as the concentrations desired for high-quality food production are far below the action levels. Unfortunately, the background concentrations of trace metals for Central and Eastern European Chernozems have not been approximated, or approximations have been made based on parent material (loess) only (Kabata-Pendias 2004).

The aim of this study was to estimate the baseline values of trace elements in modern Chernozems developed from loess and to compare these with metal concentrations in the buried Chernozems, as “natural” geochemical background, and then check the following hypotheses: (1) there is no statistically significant difference between the simple statistical measures (mean and median) for background and the refined measures derived using the iterative 2σ technique in the Chernozems located in uncontaminated areas; (2) baseline values derived for parent material cannot be applied to topsoil layers, i.e., topsoil layers require separate baseline values; (3) the anthropogenic accumulation of trace metals may be distinguished from natural bioaccumulation in the thick humus horizons of Chernozems; and (4) the impact of naturally enhanced background may be distinguished from anthropogenic contamination. The findings will be important for the monitoring of apparent soil contamination with trace metals, and this will provide an appropriate basis for risk assessments for high-quality food production on the world’s most valuable soils—Chernozems developed from loess.

## Materials and methods

### Area of study

**Fig. 1:**
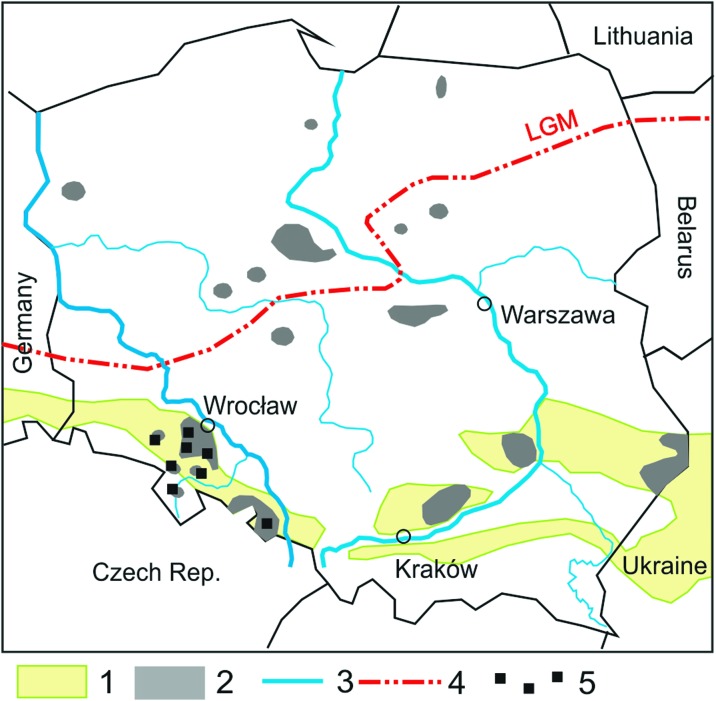
Distribution of “chernozems” and “black earths” in Poland in relation to loess covers. Soil contours based on Mocek (2015), loess contours based on Jary (2007). Explanation: 1, loess; 2, czernozems (dry) and black earths (moist); 3, main rivers; 4, Last Glacial Maximum (LGM) extend; 5, study site. For interpretation of the references to color in this figure legend, the reader is referred to the web version of this article

The study was conducted in SW Poland, within the so-called loess belt extending from Russia and Ukraine, through southern Poland and toward Germany (Fig. 1). Loess covers in SW Poland mainly occur in the Silesian Lowland and the Sudeten Foreland, which are extensive flat regions, more undulating/hilly in the southern part, in the transition to the Sudeten Mountains. The contemporary relief of the region was formed by two/three Pleistocene glaciations and subsequent weathering and denudation processes. Among the glacial deposits in SW Poland, ground (bottom) moraine tills and the glacio-fluvial sands prevail. The last glaciation (Vistulian) was the key period for relief shaping and soil cover development due to widespread loess accumulation (Badura et al. 2013). The thickest loess sediments (more than 3 m thick), with preserved original sedimentary structures, primarily occur at the borders of the Silesian Lowland. In the central part of the Silesian Lowland and the Sudeten Foreland, the loess cover is shallower, in many places < 1 m thick, and in some sites it is completely degraded. It cannot be excluded that, during the strong eolian action and formation of loess covers, as well as during the post-sedimentary slope processes, loess may have received admixtures of allogenic Pleistocene or older materials derived, for example, from the local outcrops of granite, gabbro, or serpentinite regoliths (Waroszewski et al. 2018). These types of admixtures may have influenced the texture of some loess patches, and also their mineralogical and chemical compositions, which is particularly likely in the case of serpentinite admixture.

Haplic/Stagnic Luvisols, in mosaics with Eutric Cambisols, Stagnosols, and Gleysols (IUSS Working Group WRB 2015), dominate among the soils developed from loess in SW Poland (Kabala et al. 2015). However, soils with thick humus horizons that satisfy the criteria for a diagnostic mollic/chernic horizon are common or locally prevail in the central part of the region. According to WRB (IUSS Working Group WRB 2015), these soils are classified as Chernozems (well-drained soils) or Phaeozems, often with Gleyic/Stagnic qualifiers (Labaz et al. 2018).

The area of SW Poland has a mild, temperate climate, with a mean annual air temperature of about 9.5 °C, a mean annual precipitation of 500–650 mm, and a growing season lasting about 225–235 days. The mild climate and fertile silt-loamy soils are conducive to intensive agriculture in this region. Currently, wheat, maize, barley, oilseed rape, and, locally, also sugar beet dominate among the crops (Labaz et al. 2018).

Both the Silesian Lowland and the Sudeten Foreland have been locally occupied by humans since the Paleolithic period and were permanently settled by humans in the Neolithic period (about 6500 bp) due to their favorable topographical, climatic, and soil conditions. A dense settlement network contributed to the early and extensive deforestation, and the spread of pastures and arable lands.

### Field and laboratory methods

The survey was carried out in SW Poland (the Silesian Lowland and the Sudeten Foreland) and included 28 profiles of arable soils classified as Chernozems or Phaeozems (IUSS Working Group WRB 2015). According to the Polish Soil Classification (2011), the soils are Chernozems (well-drained soils) and Black Earths (soils with Gleyic/Stagnic properties). Soil pits were dug to a depth of 120–160 cm, to reach unaltered parent material. The majority of the soil profiles were developed from thick loess covers (> 200 cm); thus, the soils have homogeneous textures of silt loam throughout the profiles, and the topsoil horizons are developed from the same material as all other genetic horizons (the soils are designated group Ch1 throughout the text and in tables/figures). In 11 soil profiles, the topsoil and subsoil horizons are developed from loess, while the bottom C horizons are developed from other underlying materials, mostly glacio-fluvial sands or glacial loams. Thus, in this group of soils, the material present in the C horizon cannot be considered the parent material for topsoil layers (soil group designated Ch2). During the laboratory analyses, a particularly high nickel content was found in some soils developed from loess. These soils are located close to an outcrop of serpentinite rocks, regoliths which were probably blown out during the periglacial eolian activity (in the Vistulian period) and were sources of a specific admixture to the silty material, transported by wind from the glacier forefields or from the glacial Odra Great Valley (Badura et al. 2013). These soils were distinguished as a separate group—Ch3. Moreover, the buried Chernozem developed from loess and discovered within the Neolithic earthen barrow (kurgan) in Muszkowice near Henrykow was taken as a “natural reference soil” for the surface Chernozems (Kabala et al. 2015).

Soil samples were collected from all genetic horizons and then, based on their original designation, were technically allocated to groups of horizons to allow reliable statistical analysis. Topsoil arable layers (unified designation—Ap) that meet the criteria for mollic/chernic horizons have a similar thickness of about 30 cm throughout the entire area. The subsurface (non-plowed) part of the humus horizons (unified designation—A2) also meet the criteria for the diagnostic mollic horizon and usually reach the depth of 50–60 cm or more. The transitional horizons are variable and had originally been described as AC, AB, Bw, Bt, or BC, taking into account the pedogenic features. All these transitional horizons were grouped together and given the unified designation B (to enable statistical analysis). However, two B layers were distinguished in group Ch2 to reflect the lithological differences between transitional layers in these soils: an upper B1 (silt-loam textures) and an underlying B2 (comprising non-silty textures). The layers of parent material have the unified designation C (regardless of the presence or absence of carbonates; it was initially proven that the presence of secondary carbonates does not differentiate the C and Ck genetic horizons in terms of trace metal content). However, the morphology, texture, and physico-chemical properties of Ap, A1, and B1 horizons in soils developed from loess (silt-loam textured) in groups Ch1 and Ch2 are very similar; thus, the horizons were respectively merged for further statistical calculations. Finally, in soil groups Ch1 and Ch2 (Tables 1 and 2), the joint values for Ap, A1, and B1 horizons are displayed, whereas the values for non-silty B2 (in group Ch2) and for C horizons (silty textured in group Ch1, and non-silty textured in Ch2) are displayed separately. The data for group Ch3 are given in a separate table, irrespective of their textural similarity to Ch1, due to the above-described specifically high concentration of nickel (Table 3).

**Table 1:** Texture and basic physicochemical properties of Chernozems in SW Poland (groups Ch1 and Ch2 as explained below)

Soil horizon	Parameter	Particle-size distribution								pH H_2_O		CaCO_3_		TOC		N_t_		BC	
		> 2.0		2.0–0.05		0.05–0.002		<0.002				%						cmol(+) kg^−1^	
		%																	
Ch1 + Ch2																			
Ap (*n* = 24)	Mean	0^a^		18^a^		68^a^		14^ab^		6.8^a^		0.15^a^		1.66^a^		0.17^a^		17^a^	
	Median	0		19		67		14		6.8		0.10		1.58		0.16		16	
	Range	0–5.3		10–38		57–83		3–24		5.3–8.7		0–1.6		0.8–2.6		0.1–0.2		9.9–44	
	SD	1.14		7.3		6.8		4.6		0.78		0.36		0.44		0.04		7.1	
A1 (*n* = 21)	Mean	0^a^		16^a^		67^a^		17^b^		7.2^a^		0.20^a^		1.23^a^		0.12^a^		18^a^	
	Median	0		15		68		17		7.2		0.10		1.22		0.12		15	
	Range	0–5.0		6–40		48–77		10–30		6.3–8.0		0–1.3		0.6–1.96		0.08–0.15		7.0–46	
	SD	1.16		8.4		8.4		5.1		0.54		0.42		0.35		0.02		9.5	
B1 (silt) (*n* = 40)	Mean	0^a^		18^a^		65^a^		18^b^		7.8^ab^		1.4^ab^		0.24^b^		–		26^ab^	
	Median	0		17		65		19		7.8		1.6		0.22		–		27	
	Range	0		8–35		54–73		9–24		6.5–8.8		0–18.9		0.1–0.7		–		7.6–86	
	SD	0		0.7		5.0		4.0		0.55		3.56		0.14		–		24	
		Ch1	Ch2	Ch1	Ch2	Ch1	Ch2	Ch1	Ch2	Ch1	Ch2	Ch1	Ch2	Ch1	Ch2	Ch1	Ch2	Ch1	Ch2
B2 (non-silty)	Mean	–	27^c^	–	60^b^	–	27^b^	–	13^ab^	–	7.8^ab^	–	3.1^ab^	–	0.18^b^	–	–	–	31^ab^
(*n* = 5)	Median	–	11	–	61	–	25	–	14	–	7.8	–	2.5	–	0.14	–	–	–	32
	Range	–	0–63	–	26–92	–	2–51	–	6–23	–	7.2–8.4	–	0–7.1	–	0.1–0.5	–	–	–	3.9–66
	SD	–	29.8	–	24.4	–	19.4	–	6.3	–	0.51	–	3.16	–	0.19	–	–	–	25
C/Ck	Mean	0^a^	8^b^	13^a^	66^b^	68^a^	25^b^	18^b^	9^a^	8.0^b^	7.8^ab^	6.3^b^	2.0^ab^	0.23^b^	0.18^b^	–	–	57^c^	27^ab^
Ch1 (*n* = 18)	Median	0	3	13	65	70	26	17	9	8.0	7.8	5.7	1.6	0.18	0.12	–	–	68	22
Ch2 (*n* = 11)	Range	0	0–34	8–22	24–97	60–73	2–61	14–24	1–21	7.4–8.4	7.0–8.6	0–16.4	0–5.8	0.1–0.7	0–0.5	–	–	11–90	2–63
	SD	0	27.6	3.55	6.45	3.88	2.11	3.08	6.45	0.32	0.56	5.14	2.11	0.20	0.14	–	–	27	22

Ch1—Chernozems with homogeneous silt-loam texture through the soil profile (i.e., in all horizons Ap-A1-B1-C/Ck); Ch2—Chernozems with silt-loam texture in topsoil (in horizons Ap-A1-B1) and non-silty textures in underlying horizons B2-C/Ck; TOC—total organic carbon; N_t_—total nitrogen; BC—sum of base cations; SD—standard deviation. Superscript letters ^a b c d^ indicate homogeneous groups of means as proven using Tukey test, i.e., the same letter indicates no statistical difference between mean values in subsequent soil horizons (tested separately for all soil properties). Values are merged and displayed together for silty topsoil horizons of soil groups Ch1 and Ch2, whereas for subsoil horizons, B2 and C/Ck are presented separately for groups Ch1 and Ch2 that differ in texture

**Table 2:** Iron and trace metal concentrations in soil horizons of Chernozems in SW Poland (groups Ch1 and Ch2 as explained below)

Soil horizon	Parameter	Fe		Mn		Zn		Cd		Pb		Cu		Ni	
		%		mg kg^−1^											
Ch1 and Ch2															
Ap	Mean	1.88^a^		544^a^		50.7^a^		0.28^a^		17.8^a^		14.5^a^		27.2^a^	
(*n* = 24)	Median	1.89		537		48.5		0.26		17.3		14.3		26.1	
	Range	1.4–2.5		276–871		40.5–96.0		0.2–0.4		13.8–23.1		10.5–19.0		13.6–77.1	
	SD	0.30		130		11.4		0.08		2.2		2.1		13.2	
A1	Mean	1.81^a^		459^a^		39.6^ab^		0.22^a^		12.8^a^		13.2^a^		19.7^ab^	
(*n* = 21)	Median	1.70		465		40.0		0.20		13.3		13.0		19.0	
	Range	1.5–2.6		122–718		31.0–50.0		0.1–0.4		7.8–18.4		6.5–17.5		13.5–30.4	
	SD	0.33		174		4.4		0.13		3.7		2.7		5.4	
B1	Mean	2.00^a^		375^ab^		36.0^ab^		0.18^ab^		8.9^b^		11.4^ab^		20.3^ab^	
(*n* = 40)	Median	2.00		388		37.0		0.15		8.8		11.5		20.4	
	Range	1.2–3.1		112–646		23.0–47.0		0.1–0.4		5.8–13.6		5.5–17.0		11.1–30.5	
	SD	0.36		112		4.8		0.10		1.5		2.1		3.4	
		Ch1	Ch2	Ch1	Ch2	Ch1	Ch2	Ch1	Ch2	Ch1	Ch2	Ch1	Ch2	Ch1	Ch2
B2 (non-silty)	Mean	**–**	1.40^b^	–	373^ab^	–	28.3^b^	–	0.13^b^	–	7.2^b^	–	7.3^b^	–	15.1^ab^
(*n* = 5)	Median		1.37		342		28.5		0.11		7.4		7.2		14.7
	Range	–	1.1–1.7	–	103–761	–	27.0–30.0	–	0.1–0.3	–	5.6–8.9	–	2.0–16.0	–	10.1–18.1
	SD	–	0.25	–	277	–	1.3	–	0.05	–	1.5	–	4.6	–	3.1
C/Ck	Mean	1.69^a^	0.99^b^	343^b^	335^b^	31.7^b^	23.5^b^	0.13^ab^	0.10^b^	7.8^b^	6.2^b^	9.5^b^	6.9^b^	16.4^b^	10.6^b^
Ch1 (*n* = 18)	Median	1.67	0.90	325	293	32.3	23.8	0.10	0.09	7.9	6.2	8.8	7.0	15.7	9.3
Ch2 (*n* = 11)	Range	1.4–2.2	0.2–2.6	161–798	32–718	26–38	10.5–39.5	0.05–0.3	0.06–0.2	5.7–11.5	2.4–11.7	7.5–14.0	4.0–8.5	10.8–22.0	2.0–21.5
	SD	0.22	0.73	152	287	4.0	10.4	0.06	0.04	1.6	3.1	1.7	1.9	3.6	6.9

Explanation: as in Table 1

**Table 3:** Texture, basic physicochemical properties, and metal concentrations in a group Ch3 of Chernozems in SW Poland (soils featured by serpentine bedrock influences)

Soil horizon	Parameter	Particle-size distribution				pH H_2_O	CaCO_3_	TOC	N_t_	BC	Fe	Mn	Zn	Cd	Pb	Cu	Ni
		> 2.0	2.0–0.05	0.05–0.002	< 0.002		%			cmol(+) kg^−1^	%	mg kg^−1^					
		%															
Ap	Mean	0	16^a^	67^a^	18^a^	6.4^a^	0.6^a^	1.49^a^	0.14^a^	20.8^a^	1.74^a^	433^a^	49.1^a^	0.24^a^	16.5^a^	15.0^a^	36.0^a^
(*n* = 10)	Median	0	16	66	19	6.2	0.2	1.43	0.14	13.2	1.75	424	49.3	0.21	16.5	14.8	33.6
	Range	0	10–25	61–75	8–25	5.5–7.0	0–5.1	1.1–2.0	0.1–0.2	11.0–72.5	1.5–1.9	319–562	46–52	0.1–0.3	12.0–19.1	12.5–18.0	29.7–45.0
	SD	0	5	4	5	0.8	1.6	0.3	0.04	19	0.13	77	2.1	0.05	2.4	1.94	5.1
A1	Mean	0	16^a^	66^a^	19^a^	7.2^ab^	2.0^b^	1.36^a^	0.12^a^	31.2^a^	1.77^a^	470^a^	44.1^ab^	0.18^ab^	12.5^ab^	14.5^a^	35.5^a^
(*n* = 13)	Median	0	17	65	19	7.4	0.3	1.41	0.14	16.4	1.75	447	44.0	0.15	11.5	14.5	37.8
	Range	0	9–31	51–77	13–27	6.1–8.0	0–17.3	0.8–1.9	0.1–0.2	12.7–93.7	1.6–2.0	177–806	36.5–49.0	0.1–0.3	7.6–21.1	10.0–17.5	26.4–41.6
	SD	0	7	7	4	0.6	4.8	0.3	0.03	29	0.14	163	3.4	0.07	3.5	2.02	4.9
B1	Mean	0	23^a^	61^a^	17^a^	7.6^ab^	3.2^b^	0.39^b^	n.o.	33.9^a^	1.70^a^	353^ab^	37.5^b^	0.13^b^	8.85^b^	10.8^b^	39.5^a^
(*n* = 20)	Median	0	19	64	18	7.8	0.4	0.36	n.o.	19.9	1.66	348	34.0	0.10	7.78	10.0	40.3
(*n* = 20)	Range	0	10–53	37–73	8–25	6.3–8.1	0–17.7	0.2–0.7	n.o.	11.6–81.5	1.3–2.1	170–761	30.5–47.5	0.1–0.4	5.7–14.6	8.5–16.0	0.4–51.6
	SD	0	13	9	5	0.5	4.9	0.2	n.o.	26	0.25	123	5.5	0.09	2.7	2.05	18.3
C	Mean	0	14^a^	70^a^	16^a^	7.8^b^	4.3^b^	0.19^b^	n.o.	43.8^a^	1.67^a^	315^b^	36.9^b^	0.11^b^	6.94^b^	10.0^b^	32.7^a^
(*n* = 7)	Median	0	13	70	17	7.8	4.2	0.15	n.o.	51.6	1.65	313	35.1	0.09	6.83	9.1	31.9
	Range	0	11–21	67–74	9–20	7.6–8.0	0–11.6	0.1–0.5	n.o.	11.5–75.1	1.3–1.9	200–431	33.0–42.5	0.1–0.2	5.9–11.9	9.5–12.0	24.5–40.6
	SD	0	3	2	4	0.2	4.8	0.1	n.o.	30	0.23	75	4.4	0.06	2.1	0.91	6.3

Explanation: as in Table 1

Moreover, samples from the A and C horizons (four samples of each) of a Chernozem buried under the Neolithic kurgan (earthen barrow) discovered near the village Henrykow (central part of the loess belt in SW Poland) have been collected to analyze the concentration of trace metals. Basic physicochemical properties of the central profile of the buried Chernozem were published previously (Kabala et al. 2015).

Soil samples were dried, crushed, and sieved to separate the skeletal fraction (> 2 mm), if present. The following analyses were performed on the fine earths (< 2 mm): particle-size distribution using sieves (sand fraction) and the hydrometer method (silt and clay fractions) after sample dispersion with Na-hexametaphosphate; soil pH in distilled water, potentiometrically, at the soil/liquid ratio 1:2.5 (*v*/*v*); calcium carbonate equivalent (CaCO_3_) by the volumetric method using a Scheibler apparatus (Van Reeuwijk 1992); total organic carbon (TOC)—by dry combustion with spectrometric measurement of the released CO_2_, using a Ströhlein CS-mat 5500 automated analyzer (after carbonate removal); total nitrogen (N_t_)—by the Kjeldahl method using a Büchi semiautomated analyzer; and the sum of exchangeable base cations (BC) extracted with 1 M ammonium acetate and analyzed by inductively coupled plasma (ICP-AES Thermo Scientific iCAP 7400). Pseudo-total concentration of iron and trace elements (Cd, Cu, Mn, Pb, Zn), after extraction with “aqua regia” (concentrated HCl/HNO_3_, 3:1), was measured by the ICP-AES technique (Thermo Scientific iCAP 7400) in a certified geochemical laboratory. The accuracy and precision of trace metal measurements were assured by sample analysis in triplicate and regular blank sample controls, as well as involvement of the samples with certified concentrations of metals under analysis (CRM materials).

Basic statistical characteristics, i.e., mean, median, and standard deviation, were extended by Pearson correlation coefficients (at *p* < 0.05) and principal component analysis (PCA) to test the relationships between metal content and soil properties. The Fisher post hoc test was applied to evaluate the statistical significance of differences between the derived mean values. To approximate the ambient background upper concentration limits (expected maximum), two kinds of threshold were calculated. The first of these was the sum of medians and median absolute deviations (Med + 2MAD). The other method approaches a normal range of data by iterative elimination of outliers, i.e., values beyond the mean ± 2 standard deviation range, further abbreviated as *x* + 2SD (Gałuszka 2007; Matschullat et al. 2000). For particular metals/soil layers, this range was approached in one to five steps. On the other hand, relative indexes of metal concentration in the topsoil were also calculated, i.e., (1) the ratio of metal concentration in the plow layer (Ap) to the directly underlying, non-plowed humus subhorizon (A1), and (2) the modified geoaccumulation index (*I*_geo_), considered to evaluate topsoil contamination in relation to bedrock geochemistry, but taking into account the pedogenic processes (Kowalska et al. 2016; Müller 1969), and calculated using the following formula:

$$ {I}_{\mathrm{geo}}={\log}_2\ \left(A/1.5\times C\right), $$

where:

*I*_geo_: geoaccumulation index
*A*: metal concentration in topsoil (plow horizon)
*C*: metal concentration in parent material (C horizon).

All statistical analyses were performed using the Statistica 13 software package (StatSoft Inc.).

## Results

**Fig. 2:**
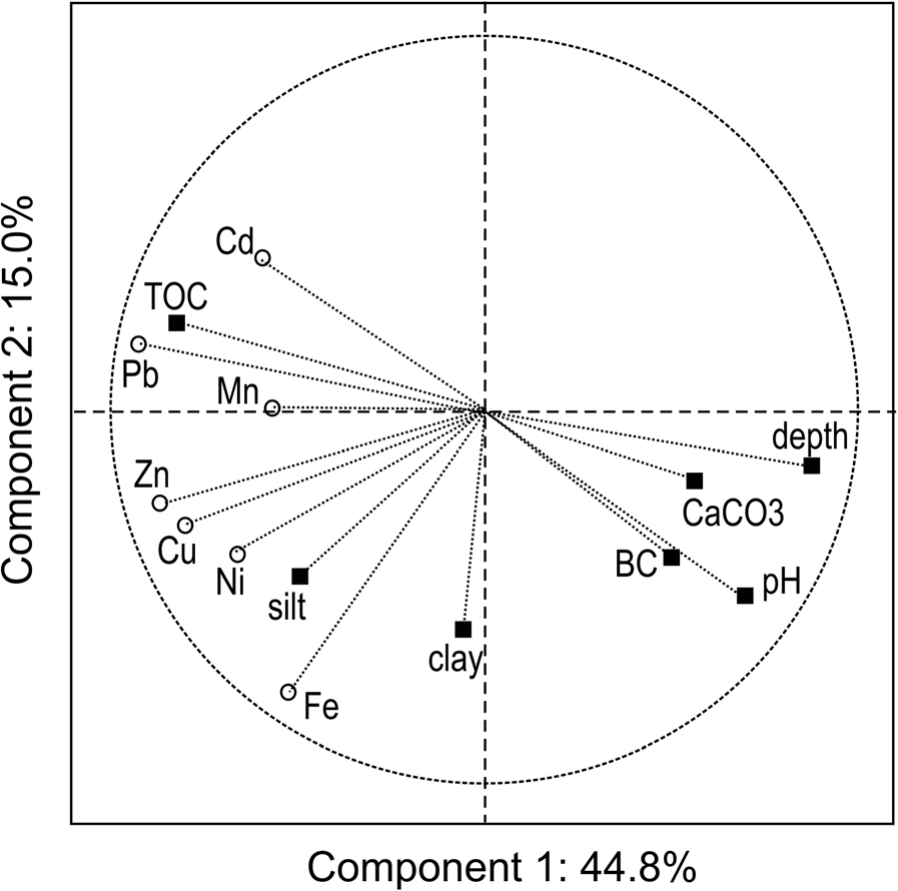
Principal component analysis for the depth in soil profile, basic soil properties, and the concentration of Fe and trace elements in Chernozems developed from loess (groups Ch1 and Ch2, as explained in Table 1). BC, sum of base cations; TOC, total organic carbon; depth, sampling depth in soil profile; clay, fraction <0.002; silt, fraction 0.05–0.002

**Fig. 3:**
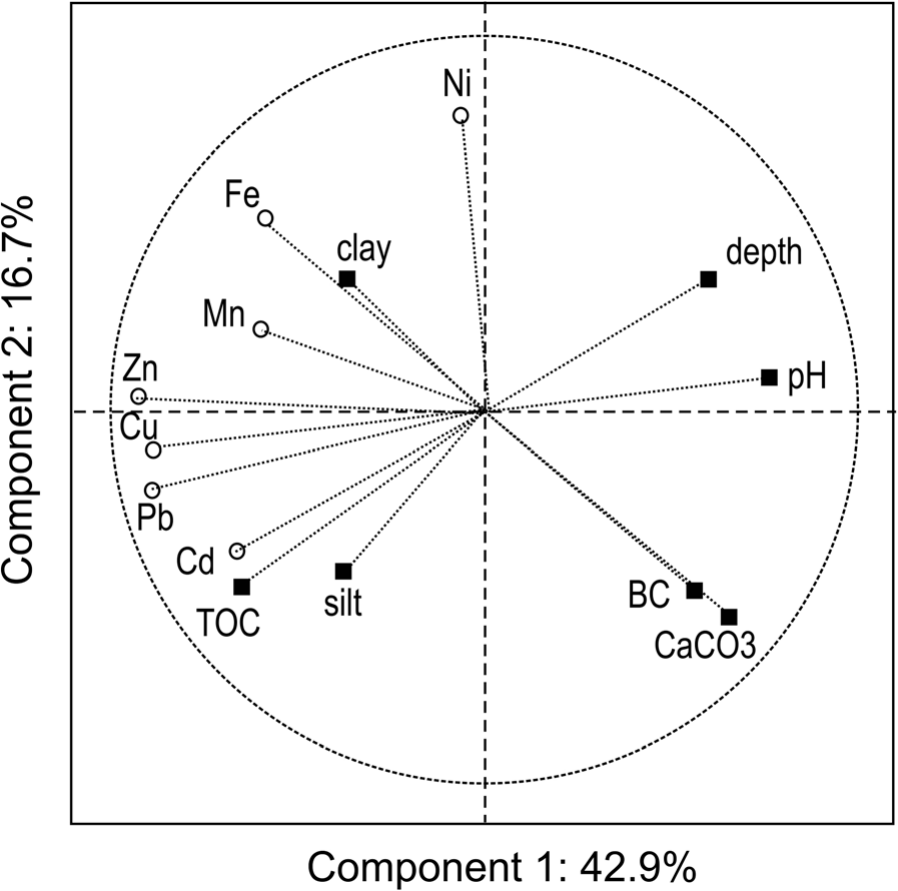
Principal component analysis for the depth in soil profile, basic soil properties, and the concentration of Fe and trace elements in Chernozems developed from loess with significantly enhanced concentration of nickel (group Ch3 as explained in Table 1). Explanation of abbreviations as in Fig. 2

Chernozems where the entire profiles are developed from loess (groups Ch1 and Ch3) and also the upper layers of Chernozems in the Ch2 group (Table 1) have typical properties for loess-derived Chernozems in a temperate climate zone. Those soils do not contain a skeletal fraction (> 2 mm in diameter), have a dominant silt fraction among the fine earth fractions (66% on average), a sand content of up to 20% (mean value), and a clay content of 12–19% (Tables 1 and 3). This particle-size distribution gives a texture class of silt loam in all layers developed from loess. Other texture classes (sands, less often loams) were only identified in the non-loess B2 and C horizons, which underlay the loess in the Ch2 profiles (Table 2). All soils have neutral or near neutral reactions in topsoil and alkaline reactions in subsoil horizons (pH increases with depth within soil profiles), which is related to the presence of carbonates. The content of calcium carbonate increases with depth, up to 16–19% (Tables 1 and 3), which also entails a significant increase in exchangeable base cations with depth, in particular calcium (Figs. 2 and 3). Such a distribution of pH values and CaCO_s_, typical for many Chernozems existing under more humid temperate climates, is related to downward leaching of carbonates that leads to depletion of the topsoil with base cations and its acidification, and then to humus and clay eluviation (Chendev et al. 2017). The soils under study are rich in organic carbon throughout the whole humus horizon (Ap + A1), 1.24–1.66% on average, while the organic carbon content decreases significantly in transitional horizons and then in C horizons (Tables 1 and 3). The high nitrogen content in Ap and A1 horizons (0.12–0.17% on average), as well as the narrow C/N ratio (10–11:1), confirms the high intensity of farming (mineral fertilization), but also high level of biological activity of the topsoil layers.

The median was in most cases slightly lower than the mean concentration of metal in a particular soil layer (0–5% of relative difference). The median was 10–20% lower than the mean concentration only in the case of cadmium only (Tables 2 and 3).

The concentration of all elements was the highest in topsoil horizons (Ap and A1) and the lowest in the parent rock layers (C/Ck), but the vertical differences between soil layers and the statistical significance of these differences are highly variable (Tables 2 and 3). In the homogeneous, loess-derived Chernozems (group Ch1), the least vertical variability was found in the case of iron (no statistically significant differences between the soil horizons). The contents of Mn, Zn, Cu, Ni, and Cd in the Ap (arable) horizon were 1.5–2 times higher than in the parent rock (C horizon), and in the case of Pb—more than twice as high (Table 2); and in all cases (but not for Cd), the difference between Ap and C was statistically significant. In group Ch2, where a lower content of elements in the sandy subsoil horizons is presumed, the ratio of element contents in Ap and C horizons exceeded the value of 2 (including Fe), except for Mn, whose concentration also decreased with depth, but to a lesser extent compared to other metals (Table 2).

The mean concentrations of Fe, Mn, Zn, Pb, and Cu in particular layers of the homogeneous, loess-derived Chernozems of the Ch1 group did not differ from their concentrations in the respective horizons of the Ch3 group (Tables 2 and 3). Only the Ni concentrations in all soil horizons of the Ch3 group are definitely higher than in the Ch1 group, starting from the parent rock horizon, where 32.7 and 16.4 mg Ni/kg were found in groups Ch3 and Ch1, respectively (Tables 2 and 3). The concentration of Ni in Chernozem profiles of the Ch3 group slightly increases toward the topsoil horizons, but the differences are statistically insignificant (Table 3). The vertical distribution of other elements was similar to described in profiles of the Ch1 group, i.e., Fe concentration did not differ throughout the profile while the concentrations of Mn, Zn, Cd, Pb, and Cu significantly increased from the parent material layer toward the topsoil (Table 3).

The difference in element concentrations between Ap and A1 layers seems to be crucial for an understanding of the effects of natural bioaccumulation and anthropogenic pollution on the concentration of elements. In the loess-derived Chernozems, the relative differences (the ratio Ap/A1) decreased from 1.4 to 1.04 in the following order: Pb = Ni > Zn > Cd > Mn > Cu > Fe (Table 2). In the Ch3 group (Table 3), the order is somewhat different—Cd = Pb > Zn > Cu > Ni > Fe > Mn—and the ratio Ap/A1 decreased from 1.3 to 0.92, respectively.

Statistical analysis, including PCA, verified the strength of the dependence between metal content and soil properties. The relationship between the metal concentration in a particular soil layer and the depth of this layer’s location within a soil profile was confirmed; however, the relationship in all soils is very weak in the case of iron (Figs. 2 and 3, Tables 4 and 5), and also insignificant in the case of nickel in the Ch3 group (Fig. 3). Generally, the clay fraction did not affect the metal distribution in the profiles of the Chernozems under study, except for iron (Figs. 2 and 3). The strong relationship between the concentration of trace elements and TOC, confirmed for Pb, Cd, Cu, and Zn (Tables 4 and 5), must be analyzed simultaneously with the similarly strong dependence on the depth in the soil profile (Figs. 2 and 3). The contents of Zn, Cu, Pb, and Mn were strongly mutually correlated in all soils (Tables 4 and 5), which means that the same factors and mechanisms jointly regulate the concentration of the group of trace elements, in particular their concentration in topsoil layers, regardless of the content of TOC and clay fraction (Figs. 2 and 3). The relationship between trace elements and Fe is weak or statistically insignificant. In “normal” loess-derived Chernozems (group Ch1 and topsoil of group Ch2), the concentration of Ni is mutually correlated with the other trace elements (Fig. 2), whereas in the Ch3 group it does not correlate either with the concentrations of other metals, or with the depth in the soil profile, or with the physico-chemical properties of the soil (Fig. 3).

**Table 4:** Coefficients of correlation between depth, soil properties, and concentration of iron and trace metals in Chernozems of SW Poland (calculated for combined groups Ch1 + Ch2, *n* = 113)

Variable	Fe	Mn	Zn	Cd	Pb	Cu	Ni
Depth	− 0.15	− 0.41*	− 0.70*	− 0.56*	− 0.82*	− 0.63*	− 0.47*
Sand fraction	− 0.55*	− 0.13	− 0.37*	− 0.27*	− 0.34*	− 0.46*	− 0.27*
Silt fraction	0.50*	0.17	0.40*	0.28*	0.44*	0.49*	0.29*
Clay fraction	0.45*	− 0.07	0.07	0.09	− 0.15	0.14	0.06
pH	− 0.02	− 0.37*	− 0.39*	− 0.70*	− 0.70*	− 0.34*	− 0.19*
CaCO_3_	− 0.28*	− 0.23*	− 0.32*	− 0.16	− 0.43*	− 0.45*	− 0.32*
TOC	0.16	0.37*	0.68*	0.61*	0.80*	0.65*	0.39*
BC	− 0.11	− 0.23*	− 0.23*	− 0.27*	− 0.40*	− 0.31*	− 0.22*
Fe		0.30*	0.56*	− 0.05	0.31*	0.65*	0.61*
Mn			0.45*	0.26*	0.51*	0.43*	0.36*
Zn				0.40*	0.75*	0.70*	0.82*
Cd					0.59*	0.20*	0.17
Pb						0.68*	0.53*
Cu							0.49*

*Statistically significant at *p* < 0.05, other explanations—as in Table 1

**Table 5:** Coefficients of correlation between depth, soil properties, and concentration of iron and trace metals in Chernozems of SW Poland (group Ch3, *n* = 46)

Variable	Fe	Mn	Zn	Cd	Pb	Cu	Ni
Depth	− 0.10	− 0.25	− 0.48*	− 0.45*	− 0.54*	− 0.51*	0.13
Sand fraction	− 0.60*	− 0.23	− 0.47*	− 0.25	− 0.44*	− 0.38*	0.48*
Silt fraction	0.23	0.21	0.30*	0.26	0.41*	0.30*	− 0.65*
Clay fraction	0.84*	0.12	0.46*	0.08	0.22	0.28	0.09
pH	− 0.35*	− 0.39*	− 0.60*	− 0.52*	− 0.73*	− 0.49*	0.01
CaCO_3_	− 0.51*	− 0.41*	− 0.55*	− 0.22	− 0.50*	− 0.50*	− 0.44*
TOC	0.04	0.45*	0.57*	0.49*	0.59*	0.71*	− 0.21
BC	− 0.41*	− 0.40*	− 0.43*	− 0.06	− 0.43*	− 0.34*	− 0.27
Fe		0.38*	0.62*	0.14	0.37*	0.43*	0.23
Mn			0.52*	0.22	0.37*	0.61*	0.26
Zn				0.68*	0.84*	0.86*	0.10
Cd					0.74*	0.63*	− 0.07
Pb						0.79*	− 0.10
Cu							0.04

*Statistically significant at *p* < 0.05, other explanations—as in Table 1

## Discussion

### “Raw” versus “refined” baseline values for topsoil layers of Chernozems

Data transformation using the iterative 2σ technique, in as many as one to five subsequent steps (if necessary), did not affect the mean concentrations of the trace elements in the distinguished (unified) soil layers, as the differences between the “raw” (not transformed) mean concentrations (Tables 2 and 3) and respective “refined” means (Table 6) were not statistically significant in any case. In most cases (Table 7), the “refined” mean was either closer to the “raw” median than to the “raw” mean, or even identical with “raw” median. Such a situation is not unusual and usually attests to the high level of normality of the data distribution (Matschullat et al. 2000) and confirms the appropriate selection of an area to investigate geochemical background. Also, the latter finding confirms the usefulness of the simple median value, instead of more elaborated indices, to characterize the geochemical baseline in appropriately selected areas, where the element concentration has a distribution close to a normal model.

**Table 6:** “Refined” mean values (mean_r_) and threshold values (“ambient background threshold”) for iron and trace metals in Chernozems of SW Poland

Soil horizon	Parameter	Fe		Mn		Zn		Cd		Pb		Cu		Ni	
		%		mg kg^−1^											
		Ch1 + Ch2													
Ap	Mean_r_ (*x*)	1.85		541		47.6		0.26		17.3		14.3		24.1	
	Threshold_r_ (*x* + 2SD)	2.40		741		57.7		0.48		20.9		18.2		37.6	
	Threshold_m_ (Med + 2MAD)	2.41		699		58.5		0.46		19.7		16.7		37.2	
		Ch1	Ch2	Ch1	Ch2	Ch1	Ch2	Ch1	Ch2	Ch1	Ch2	Ch1	Ch2	Ch1	Ch2
C/Ck	Mean_r_ (*x*)	1.67	0.89	303	345	31.7	25.0	0.12	0.10	7.55	6.25	9.21	6.90	16.4	11.5
	Threshold_r_ (*x* + 2SD)	2.05	1.93	484	889	39.7	47.3	0.20	0.20	10.3	12.2	11.9	10.7	23.6	24.4
	Threshold_m_ (Med + 2MAD)	2.03	1.76	491	735	40.3	47.0	0.18	0.19	10.1	10.0	11.3	9.7	21.8	22.4
Ch3															
Ap	Mean_r_ (*x*)	1.74		433		49.1		0.24		16.5		15.0		36.0	
	Threshold_r_ (*x* + 2SD)	1.99		587		53.3		0.36		21.4		18.9		46.1	
	Threshold_m_ (Med + 2MAD)	1.96		605		48.0		0.31		20.0		15.9		41.0	
C/Ck	Mean_r_ (*x*)	1.67		315		36.5		0.11		6.90		9.8		32.7	
	Threshold_r_ (*x* + 2SD)	2.14		466		44.6		0.19		10.9		11.6		45.3	
	Threshold_m_ (Med + 2MAD)	1.95		414		44.5		0.12		9.3		12.5		44.1	

Ch1—Chernozems with homogeneous silt-loam texture through the soil profile; Ch2—Chernozems with silt-loam texture in topsoil (horizons Ap–A1) and non-silty textures in underlying horizons C/Ck; Ch3—silt-loam textured Chernozems influenced by serpentine bedrock; threshold_r_—calculated as sum of mean_r_ + 2 standard deviations (Matschullat et al. 2000); threshold_m_—calculated as sum of median + 2 median absolute deviations (Mikkonen et al. 2017)

**Table 7:** Comparison of median, mean, and “refined” mean for iron and trace metals in Chernozems developed from loess in SW Poland and other reported values (rounded values)

Soils	Horizon	Parameter	Fe	Mn	Zn	Cd	Pb	Cu	Ni
			%	mg kg^−1^					
Chernozems of SW Poland developed from loess (present study)	Ap	Median	1.89	537	49	0.26	17	14	26
		Mean	1.88	544	51	0.28	18	15	27
		Mean_r_	1.85	541	48	0.26	17	14	24
	C/Ck	Median	1.67	325	32	0.10	8	9	16
		Mean	1.69	343	32	0.13	8	10	16
		Mean_r_	1.67	303	32	0.12	8	9	16
Buried Chernozem in SW Poland (Neolithic kurgan; present study)	A	Median	1.82	595	44	0.13	10	15	19
	C/Ck		1.61	320	35	0.08	8	11	17
Chernozems (world)^1^	Ap	Mean	–	480	65	0.44	23	24	25
Chernozems (SE Poland)^1^	Ap	Mean	–	560	62	0.38	25	19	–
Chernozems (Ukraine)^2^	Ap	Median	–	648	56	0.18	18	16	31

^1^Kabata-Pendias (2011)

^2^Klos et al. (2014)

The (“raw”) median concentrations of Zn, Pb, Cu, and Ni in the plow layers (Ap) of Chernozems under study are similar or significantly lower than the mean or median concentrations of these elements calculated previously for Chernozems around the world, SE Poland and Ukraine (Table 7). The median for Mn, although higher than the mean value in Chernozems around the Europe, is lower than the Mn contents in Chernozems of SE Poland and Ukraine. In turn, the median for Cd value is significantly higher than the median for Ukrainian Chernozems, but is significantly lower than the mean values for other Chernozems in Poland and around the world. A comparison to these archival mean/median values indicates that the median values determined for trace elements (except Cd) in the loess-derived Chernozems in SW Poland are reliable as a geochemical baseline to evaluate contamination in other Chernozems in Europe and around the world. The content of cadmium in the Chernozems of SW Poland, although much lower than the mean Cd content in global and other Polish Chernozems, is nearly 50% higher than in Ukrainian Chernozems. This difference is probably related to the long-term and intense fertilization of these soils with Cd-bearing phosphorus fertilizers and other biosolid amendments (McLaughlin et al. 1999), as any other sources of contamination with Cd are absent in the chernozemic region of SW Poland (Kabala et al. 2015). Nevertheless, the median values found in the present study for Mn and Cd are considered a reliable geochemical baseline, at least on a Central European scale (Kabata-Pendias 2011; Kobza et al. 2017; Komárek et al. 2008; Rékási and Filep 2012; Salminen and Tarvainen 1997; Spahić et al. 2018).

The threshold values, interpreted as the ambient background upper concentration limits (i.e., expected maximums), offer a more comprehensive basis for the evaluation of soil contamination, i.e., identification of the concentrations considered significantly higher than the geochemical background (Reimann and Garrett 2005). The thresholds calculated by two different methods—(1) as the sum of median plus two median absolute deviations (Mikkonen et al. 2017) and (2) as the sum of mean plus two standard deviations derived by iterative rejection of outliers (Matschullat et al. 2000)—have generally very similar values for all metals (Table 6). Relatively speaking, the largest differences between these two thresholds were for Mn (in Ch1 + Ch2 soils) and Cd (in Ch3 soils). For Zn, Pb, Cd, and Cu, both threshold values are close to or lower than the mean concentrations of these metals in topsoil horizons of other Chernozems in SE Poland and around the world (Tables 6 and 7). This confirms the particularly low content of these metals in the soils under study and the applicability of derived baselines (both median values and the aforementioned thresholds) to evaluate the contamination of other Chernozems developed of loess.

### Are the metal concentrations enhanced or “naturally low” in the topsoil layers of Chernozems under investigation?

Reliable, although rarely available, information on the “natural” geochemical background is provided by the presumably uncontaminated soils buried before the industrial era, or even in prehistorical periods. A unique possibility for such a comparison is offered by the complex of Neolithic kurgans (earthen barrows) recently discovered in the loess area near Henrykow, SW Poland (Kabala et al. 2015). The median values in Ap horizons of Chernozems under study in SW Poland are slightly higher, but do not differ statistically from those in the topsoil layer of the buried Chernozem in the case of Fe, Zn, Cu, and Ni (Table 7). In case of Mn, the values in modern Chernozems were found to be even lower than in the buried Chernozem. However, the values for Pb are 70% higher, and for Cd 100% higher compared to buried soil (Table 7). Even if this comparison has limited reliability as it includes one buried soil profile only, it confirms expected trends. The similarity of concentrations confirms the lack of contamination in “modern” soils, at least in the case of Fe, Mn, Zn, and Cu. These low values also confirm that, even presently in Europe, the “ambient” background may be very similar to or the same as the “natural” background, at least for selected elements and in selected areas, e.g., those free of local industrial sources of contamination (Gałuszka 2007; Migaszewski et al. 2010). The latter seems impossible for Pb and Cd due to the very common and worldwide soil contamination with these metals (Adriano 2001; Cannon and Horton 2009; Gorsuch et al. 2006). This cannot be linked to high native Cd and Pb concentrations in the parent material, as the loess in the C horizons of both modern and buried soil contains 2–2.5 times less Cd and Pb than the Ap horizons (Table 7).

### Applicability of rock-derived baselines to topsoil horizons

Historically, evaluation of soil contamination started with comparisons to “Clarke values,” i.e., mean metal concentrations in the Earth’s crust (Kabata-Pendias 2004). Although this approach was abandoned a long time ago, the comparison of metal content in the topsoil to its concentration in the parent material of soil (the so-called C horizon) is still quite popular in the form of various “enrichment factors” (Kowalska et al. 2016, 2018; Mazurek et al. 2017). This method has been criticized for failing to take into account the natural pedogenic processes that redistribute elements in the soil profile, in particular those related to podzolization and clay illuviation (Reimann and Garrett 2005). However, the approach has probably never been discussed and rejected for slightly leached soil, such as Chernozems, rich in humus and divalent cations that enhance the sorption and stabilization of trace elements.

As shown in the buried natural Chernozem (Table 7), significant differences between A and C horizons exist for Zn, Mn, and Cd concentrations; a slight difference was noted for Pb, and practically no difference for Fe and Ni concentrations. This means no difference for the latter elements (Pb, Ni, Fe) has developed naturally between topsoil and subsoil horizons in a soil type known for its high biological activity and expected high bioaccumulation rate related to TOC accumulation in the topsoil (Chendev et al. 2017). In turn, the higher concentration of Zn, Mn, and Cd in the topsoil of buried Chernozems was due to natural biogeochemical processes (bioaccumulation) and cannot be considered contamination. Therefore, at least the concentrations of Cd, Mn, and Zn in the parent material (C horizon) of the buried Chernozem cannot serve as a reliable background for the topsoil horizon (a homogeneous silty texture throughout the profile).

A comparison of mean and median values calculated for particular soil horizons of Chernozems in SW Poland (Table 2) indicates statistically significant differences between C and Ap horizons in the case of all metals, excluding only Fe (in the homogeneous silty soils, group Ch1, and also group Ch3, excluding Ni). As shown above (comparison with buried Chernozem), this difference cannot be considered the result of topsoil contamination alone. Moreover, group Ch2 represents soils with a lithological discontinuity within the profile, which is relatively common in Central/Northern Europe and North America. Lithological discontinuity (as in the case of loess underlain with glaciofluvial sand) is typically also connected with geochemical discontinuity and makes the direct comparison of the topsoil and subsoil layers unjustifiable. For all metals (including Fe), the mean values in A and C horizons of soils in group Ch2 significantly differ (Table 2). These statements confirm that metal levels in parent rock do not reflect the background values for topsoil horizons in many soils and for many metals; this thus argues for separate baseline values for topsoil layers to allow a reliable assessment of topsoil contamination (Reimann et al. 2012; Zhao et al. 2007).

### Distribution of metals in Chernozem profiles

The experimental data indicate relatively low mobility of major and trace elements in Chernozems (Borui et al. 2017; Chernikova et al. 2013; Felix-Henningsen et al. 2010) due to strong metal sorption by humic substances and stabilization of organic complexes under such favorable conditions as neutral soil pH, saturation with divalent cations, and medium-fine texture (Adriano 2001; Minkina et al. 2006, 2008; Šimansky and Jonczak 2016). Thus, it is not the leaching of metals that is the presumed reason for differentiation of the metal concentrations between subsequent horizons of Chernozems, but topsoil bioaccumulation and zooturbation, in particular translocation of humus-rich soil by burrowing animals such as moles and anecic earthworms. Labaz et al. (2018) found that the age of organic matter (*in fact*—the mean residence time, MRT) increased with depth in thick humus horizons of Chernozems in SW Poland by ca. 350–400 years per 10-cm-thick sublayer. This confirms an intense and relatively proportional translocation of “fresh” organic residues from the topsoil down to at least 50–60 cm (in SW Poland).

Based on the above statement, the concentration of an element should naturally decrease with depth within the thick humus horizon proportionally to the decrease in TOC, if the rate of inflow of the given element to soil is similar to TOC accumulation in the topsoil (Baize and Sterckeman 2001). Potential sources of metal inflow are release from mineral phase by weathering, bio-cycling, and bioaccumulation (uptake by roots from the subsoil and return with biomass), and contamination added with fertilizers or air-borne (Alloway 2013). Metal uptake from subsoil and its return to topsoil by plants could play a role under non-forest vegetation until the humus horizon becomes thicker than dominant plant roots. Moreover, in the case of crop plants, the metal return to soil from biocycling is reduced by its amount removed in the crop yield (Kabata-Pendias 2004).

Such a slight gradual decrease in metal concentration with depth in Chernozems of SW Poland was observed for copper—ca. 10% decrease in total concentration between subsequent horizons (group Ch1, Table 2). The differences in Mn and Zn concentrations between soil horizons are higher (10–22%), and the highest are differences in Cd and Pb concentrations that reach 25–30% of metal content (in relation to overlying horizon). The Fe and Ni concentrations in soil group Ch1 changed insignificantly or irregularly; thus, the depth trend is uncertain (Table 2) and may be due to the visible (Fig. 2 and Table 4) affinity of these metals to clay fraction, where the maximum was identified in subsurface B horizons (Table 1). The abrupt decrease in Pb and Cd concentrations between A1 and B1 horizons (Tables 2 and 3) confirms the particular affinity of these metals to organic matter (Fig. 2).

Chernozems have uniquely thick humus horizons, thicker than the plow layer, where all recent contaminations may concentrate, those from both air-borne pollution and fertilization. Thus, the simple comparison of the metal content in Ap and underlying A2 horizons may indicate the scale of relatively recent metal inflow that is still not “dissolved” by earthworm activity. The ratio of median metal concentration in Ap to A2 horizons, averaged for all soils under study (proportionally to the abundance of soil profiles in the groups Ch1 + Ch2 and Ch3), was the lowest for Fe, Mn, and Cu (value 1.03–1.06), intermediate for Zn and Ni (1.13–1.16), and the highest for Cd and Pb (values 1.35–1.36).

Metal distribution in the soil profile can be assessed with the use of the geoaccumulation index, *I*_geo_ (Kowalska et al. 2016; Müller 1969), where those values above 1 are considered to indicate topsoil contamination. The *I*_geo_ in the buried (natural) Chernozem has negative values for Fe, Ni, Pb, Zn, and Cu, and positive ones for Cd and Mn, but no higher than 0.3 (Table 8). In “homogeneous” Chernozems (silty texture throughout the profile) in the present study, Fe still has negative values for *I*_geo_, Ni, Zn, Mn, and Cu have values which are negative or near zero, whereas the *I*_geo_ for Cd and Pb reached the values of 0.5–0.8 (Table 8).

**Table 8:** Relative indices of iron and trace metals distribution/accumulation in Chernozems under study

Parameter/soils	Fe	Mn	Zn	Cd	Pb	Cu	Ni
Ratio of metal concentration in Ap to A2 subhorizons (all soils under study)	1.03	1.05	1.16	1.35	1.36	1.06	1.13
*I*_geo_ for buried (natural), loess-derived Chernozem	− 0.41	0.31	− 0.25	0.12	− 0.26	− 0.14	− 0.42
*I*_geo_ for homogeneous, loess-derived Chernozems (group Ch1)	− 0.41	0.14	0.03	0.79	0.50	0.05	0.12
*I*_geo_ for homogeneous, loess-derived Chernozems (groups Ch3)	− 0.50	− 0.15	− 0.09	0.64	0.69	0.12	− 0.51
*I*_geo_ for texturally heterogeneous Chernozems (group Ch2)	0.49	0.29	0.46	0.95	0.87	0.42	0.90

*I*_geo_—geoaccumulation index

All the aforementioned relative indices of metal distribution in soil profiles have confirmed that no topsoil enrichment can be concluded in the case of Fe, very little enrichment in case of Mn and Cu, little enrichment with Zn and Ni, and significant enrichment in the case of Cd and Pb.

To provide a contrast, the *I*_geo_ values in the Ch2 soil group should be discussed. Those “heterogeneous” Chernozems have a silty topsoil underlain by a sandy or loamy subsoil of other geological origin (Table 3). The minimum *I*_geo_ values started in these soils from 0.3 for Mn; reached 0.4–0.5 for Cu, Zn, and Fe; and the level 0.87–0.95, near the contamination threshold, for Ni, Cd, and Pb (Table 8). This comparison shows that unreasonable application of *I*_geo_ and other indices in texturally differentiated soils may lead to an incorrect evaluation of soil contamination and the overestimation of health and environmental risks (Kowalska et al. 2016; Mazurek et al. 2017).

### Specific concentration and distribution of Ni—an example of a naturally elevated geochemical background

The reason to separate off a number of Chernozem profiles as group Ch3 was the significantly higher concentration of Ni in all soil horizons, e.g., the twofold higher Ni concentration in C layers of Ch3 soils as compared do Ch1 soils (Tables 2 and 3, refers both to mean and median values). The lack of any depth trend in Ni concentration within the soil profile of Ch3 soils may suggest an internal source of this phenomenon—strong impact of parent material mineralogy (Bonifacio et al. 2010). Although nickel-bearing metamorphic rocks occur in numerous sites in SW Poland, and significant enrichment with Ni has been identified in soils directly influenced by these rocks (Kierczak et al. 2016; Pędziwiatr et al. 2018), the Chernozems under study are not underlain by serpentinites or other ultramafic rocks and do not adjoin known surface outcrops of these rocks. Ni content in these Chernozems is positively correlated with the sand fraction (Table 5). This is the only case, in the soils under study, where the metal concentration is significantly correlated with the coarse mineral fraction (Tables 4 and 5). The only reasonable explanation for this correlation relates to eolian processes during the Last Glacial Maximum (Vistulian period), i.e., blowing out the fine to medium sand particles from the regoliths of the exposed outcrops of ultramafic rocks, admixing to the other wind-transported particles (blown out from the glacier forefield or old-Odra glacial river valley; Badura et al. 2013), and finally, joint sedimentation as a loess cover. The concentrations of the other elements in the subsoil layer of Ch3 soils are very similar to those in Ch1 (Tables 2 and 3, and also Table 6) which suggests exceptional enrichment with Ni alone.

If the elevated Ni concentration in Ch3 soils has a natural (i.e., non-anthropogenic) origin, the significantly higher Ni concentrations in both Ap and C layers of the Chernozems in the Ch3 group (as compared to the respective layers of Chernozems in the Ch1 group) cannot be considered to represent contamination. As reported by Bonifacio et al. (2010), even much higher amounts of Ni in soils, but directly related to rock and regolith geochemistry, do not generate damage to plants naturally adapted to specific local geochemistry. Therefore, the refined mean and threshold Ni values (Table 6) could serve as a provisional geochemical baseline for loess-derived Chernozems impacted by ultramafic rock additions, e.g., in risk assessment procedures or analysis of soil monitoring data. It is also possible that the Ni concentrations may be similarly enhanced in the other soil types considered to be derived from loess (based on their silty texture), but having similar eolian admixtures of Ni-rich materials (Waroszewski et al. 2018). Further studies, including mineralogical investigations of the sand fraction, seem reasonable to explain the sources of enhanced concentrations of Ni (and possibly of the other trace and rare earth elements) in the soils developed from loess.

## Conclusions

Based on 28 profiles of chernozemic soils developed from loess in an agricultural region in SW Poland presumed to be free of industrial contamination, the geochemical baselines have been derived for Fe and six common trace metals using the analytical data for four standardized soil layers: Ap (plow layer), A1 (non-plow subsurface humus layer), B (transitional), and C (unaltered parent material). The median values for the plow layer (1.89% for Fe, and 537, 49, 17, 14, and 26 mg kg^−1^ for Mn, Zn, Pb, Cu, and Ni, respectively) are lower than the mean or median values reported for other Chernozems in SE Poland/Europe/world, and thus may serve as a general geochemical baseline for chernozemic soils developed from loess. The concentration of Cd, although lower than in other Chernozems of Poland and around the world, was significantly higher than in Ukrainian Chernozems and thus may serve as a local baseline only.

The median values for Fe, Cu, Mn, and Zn, typically considered the baselines for ambient geochemical background, are very close to the concentrations in the Chernozem buried under the Neolithic kurgan. This means the present concentrations of the above-listed elements in agricultural areas may still be close to their natural geochemical background. However, Pb and Cd concentrations were twofold higher than their respective values in the buried natural soil, indicating the scale of general contamination of the topsoil horizons of arable soils with these two metals. The ratio of metal concentration in the plow to underlying non-plowed humus subhorizons increased in the order Fe (value 1.03) < Mn = Cu < Zn = Ni < Cd = Pb (value 1.36) and confirmed a high recent input of Cd and Pb to the plowed topsoil horizons of arable soils that greatly exceeds the natural bioaccumulation and zooturbation rates.

Therefore, the values calculated as median plus two median absolute deviations or refined mean plus two standard deviations have the same or similar values (2.4% for Fe, and 58, 0.46/0.48, 20/21, 17/18, 37/38, and 699/741 mg kg^−1^ for Zn, Cd, Pb, Cu, Ni, and Mn, respectively) and may serve as threshold values to indicate the loess-derived Chernozems featured by excessive concentrations of these elements, i.e., potentially or apparently contaminated.

Concentrations of elements, both in the surface soils and in the buried Chernozem under study, excluding Fe, are significantly higher in topsoil (plow) layers compared to parent rock (loess in the C horizon). These findings justify the determination of separate (ambient) background baselines for topsoil horizons and their application in the evaluation of soil contamination scales. Moreover, in soils texturally differentiated, the topsoil and subsoil layers are of different geological origin and different geochemistry. In this case, the common geoaccumulation indexes that compare the element content in topsoil and (false) parent material may significantly overestimate the topsoil contamination.

Underlying or locally outcropped specific bedrocks (e.g., serpentinite rocks) may naturally enhance the total concentration of trace elements in an entire soil profile by the addition of metal-rich regolith particles during the formation of surface covers, e.g., by eolian processes under the periglacial conditions of the Pleistocene period. Such soils are naturally enriched with metals (with nickel in case of serpentinite bedrock), cannot be considered contaminated, and thus require a separate legal treatment, including separate (or individually suited) background baselines for health risk assessment procedures.
